# Network-Derived Radioresistant Breast Cancer Target with Candidate Inhibitors from Brown Algae: A Sequential Assessment from Target Selection to Quantum Chemical Calculation

**DOI:** 10.3390/md21100545

**Published:** 2023-10-19

**Authors:** Mahema Sivakumar, Sheikh F. Ahmad, Talha Bin Emran, Paola Isabel Angulo-Bejarano, Ashutosh Sharma, Shiek S. S. J. Ahmed

**Affiliations:** 1 Drug Discovery and Multi-Omics Laboratory, Faculty of Allied Health Sciences, Chettinad Academy of Research and Education, Chettinad Hospital and Research Institute, Kelambakkam 603103, Tamil Nadu, India; 2 Department of Pharmacology and Toxicology, College of Pharmacy, King Saud University, Riyadh 11451, Saudi Arabia; 3 Department of Pathology and Laboratory Medicine, Warren Alpert Medical School & Legorreta Cancer Center, Brown University, Providence, RI 02912, USA; 4 Department of Pharmacy, Faculty of Allied Health Sciences, Daffodil International University, Dhaka 1207, Bangladesh; 5 NatProLab-Plant Innovation Lab, Regional Department of Bioengineering, Tecnologico de Monterrey, Queretaro 76130, Mexico

**Keywords:** primary breast cancer, radioresistant breast cancer, protein network, brown algae, nahocol-A1

## Abstract

Despite significant progress in early detection and treatment, a few aggressive breast cancers still exhibit resistance to therapy. This study aimed to identify a therapeutic target for radioresistant breast cancer (RRbc) through a protein network from breast cancer genes and to evaluate potent phytochemicals against the identified target. Our approach includes the integration of differential expression genes from expression datasets to create a protein network and to use survival analysis to identify the crucial RRbc protein in order to discover a therapeutic target. Next, the phytochemicals sourced from brown algae were screened through molecular docking, ADME (absorption, distribution, metabolism, and excretion), molecular dynamics (MD) simulation, MM-GBSA, and quantum mechanics against the identified target. As a result of our protein network investigation, the proto-oncogene c-KIT (KIT) protein was identified as a potent radioresistant breast cancer target. Further, phytochemical screening establishes that nahocol-A1 from brown algae has high binding characteristics (−8.56 kcal/mol) against the KIT protein. Then, quantum chemical analysis of nahocol-A1 provided insights into its electronic properties favorable for protein binding. Also, MD simulation comprehends the conformational stability of the KIT–nahocol-A1 complex. Overall, our findings suggest nahocol-A1 could serve as a promising therapeutic candidate for radioresistant breast cancer.

## 1. Introduction

Breast cancer is a serious health concern that affects women worldwide. In recent decades, breast cancer has been the subject of significant research due to its wide prevalence, resistance to therapy, and recurrence [1]. Primary breast tumors are the first stage of the disease, which is characterized by the abnormal growth of cells within the breast tissue [2]. Early detection and treatment have significantly improved survival rates. Notably, some types of breast cancer, such as triple-negative breast cancer, are more aggressive and resistant to therapy. Currently, three predominant therapeutic approaches, such as radiotherapy, chemotherapy, and standard surgical treatments, are being adopted to treat breast cancer [3]. Besides surgery and chemotherapy, radiotherapy has potential in the clinical management of breast cancer. Though radiotherapy is effective, some individuals have reported developing recurrences due to the radioresistant behavior of tumor cells. Such resistance can lead to poor treatment outcomes and disease recurrence [4]. Thereby, radioresistance remains an elementary barrier to achieving the maximum efficacy of radiotherapy. Also, numerous drugs have been developed for breast cancer treatment. Most of the drugs have been hindered by their notable tendency to provoke resistance, thereby diminishing their efficacy as valuable therapeutic agents and restricting their utility for the treatment of breast cancer. Wang D et al. reported that sunitinib facilitates metastatic breast cancer spreading by inducing endothelial cell senescence and has no clinical benefit in breast cancer patients [5].

Several studies are being carried out to explore new therapeutic options to improve treatment outcomes for patients with radioresistant breast cancer. In recent years, investigations have shown the involvement of crucial genes and proteins that are associated with the pathogenesis of breast cancer, but the mechanism relating to radiosensitivity is still unclear [6]. Therefore, it is of great significance to explore therapeutic targets as well as potential drugs for patients with radioresistant breast cancer. Advances in omics technology and intensive computational analysis of omics data have led to the discovery of new biomarkers for diagnosis and drug targets for complex diseases like cancer [7], neurodegenerative diseases [8], and heart diseases [9].

Natural products from several sources have always played a significant role in cancer therapy. On the growing list of natural compounds, various unique secondary metabolites from marine habitats have received extensive and significant attention and have become potential leads in drug discovery due to their vast and wide biodiversity [10]. Researchers have explored many marine resources to develop novel, biologically active scaffolds for the production and development of therapeutic drugs. Marine-derived bioactive compounds have a wide range of biological activities, including anti-cancer, anti-metastatic, and cytotoxic properties, as well as antioxidant, anti-thrombotic, anti-coagulant, anti-inflammatory, anti-hypertensive, anti-diabetic, and cardio-protective activities [11]. Notably, brown algae show good biological activities, such as anti-cancer, anti-viral, anti-inflammatory, anti-proliferative, anti-cholesterol, and anti-diabetic properties. Brown algae are also useful in different conditions such as hypothyroidism and asthma. For instance, Kim et al. found that fucoidan from brown algae activates caspases via mitochondria- mediated and receptor-mediated pathways to inhibit growth and induce apoptosis in HT-29 human colon cancer cells [12]. Similarly, Alim et al. reported that fucoidan sulfated polysaccharide sourced from brown algae (*Sargassum* sp., *Turbinaria* sp. and *Padina* sp) demonstrates anti-cancer activity against human colon and breast cancer cell lines (MCF-7) with high cytotoxic activity [13]. Thus, we focused on screening the phytochemicals of brown algae as possible drug candidates against radioresistant breast cancer (RRbc).

**Figure 1:**
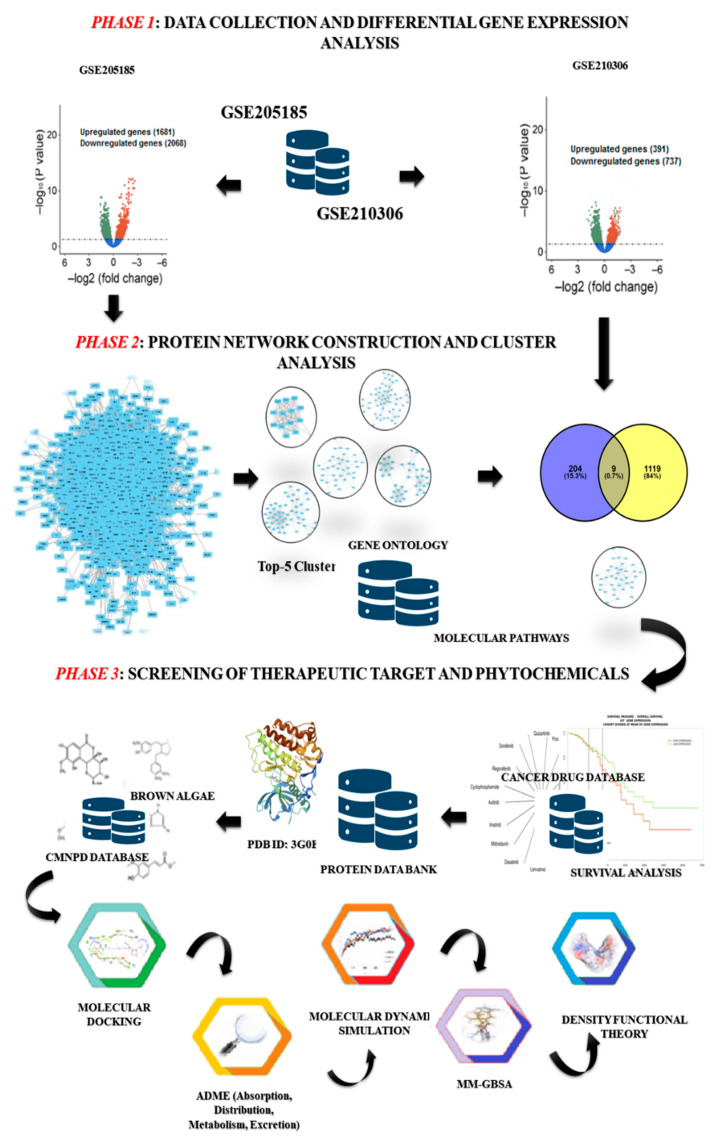
The computational workflow describes the strategies for identification of RRbc drug targets and screening of potential phytochemicals derived from brown algae. Phase 1: collection of gene expression data (primary breast cancer: GSE205185; and radioresistant breast cancer: GSE210306) and execution of differential gene expression analysis using the LIMMA R package. Phase 2: build a protein network from the differentially expressed genes of GSE205185. Then the MCODE plug-in Cytoscape was used to find the five most important clusters. Further, these clusters were mapped with the DEGs of GSE210306 to identify the overrepresented cluster with the inherited behavior of both breast cancer and radioresistant properties. Phase 3: all proteins in the selected cluster were screened for their potential as therapeutic targets and further evaluated based on survival rate. Then, the selected target was assessed against the phytochemicals sourced from brown algae through molecular docking, ADME analysis, MD simulation, MM-GBSA, and density functional theory.

Here, we have used a set of computer methods (shown in Figure 1) that combine the gene expression profiles of primary and radioresistant breast cancer (RRbc) to build a protein network and find a potential therapeutic target for RRbc. Also, we used molecular docking and ADME (absorption, distribution, metabolism, and excretion) to screen the phytochemicals of brown algae against the chosen RRbc target to find a possible drug candidate. Furthermore, a molecular dynamics (MD) simulation was performed that demonstrates the conformational stability of the protein–ligand complex, and the execution of density functional theory (DFT) provided the quantum chemical properties of the drug candidate against the protein target. In addition, molecular mechanics with a generalized Born and surface area solvation (MM-GBSA) calculation confirm the high binding affinity and stable conformation of the drug candidate with the RRbc target.

## 2. Results

### 2.1. Gene Expression Analysis of Radioresistant and Primary Breast Cancer Datasets

Two independent microarray expression profiles (GSE205185 and GSE210306) were assessed to identify the differentially expressed genes between the conditions. For instance, GSE205185 assessed the differentially expressed genes between primary breast cancer (PBC) and healthy controls, which showed 3749 DEGs, with 1681 upregulated and 2068 downregulated genes. Likewise, the assessment of GSE210306 showed 1128 DEGs, with 391 upregulated and 737 downregulated in RRbc compared with the non-radioresistant group.

### 2.2. Protein Network Construction and Target Screening

**Figure 2:**
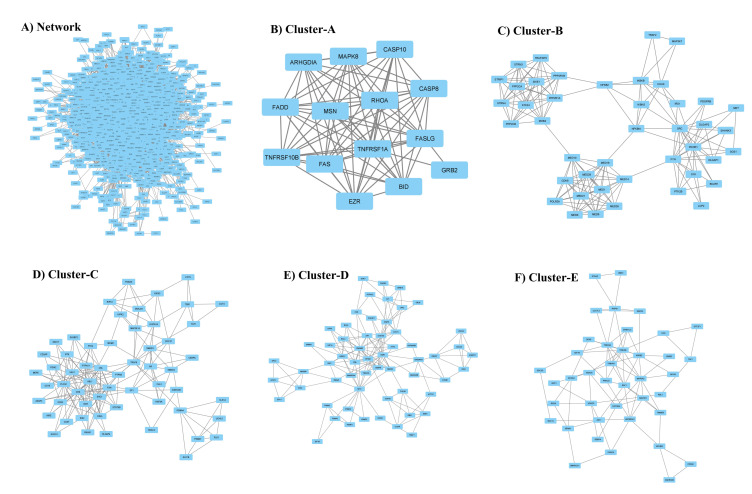
(**A**) Comprehensive protein interaction network of genes encoding proteins associated with primary breast cancer. The top five clusters (**B**–**F**) highlight the core functional segment of the protein network.

**Figure 3:**
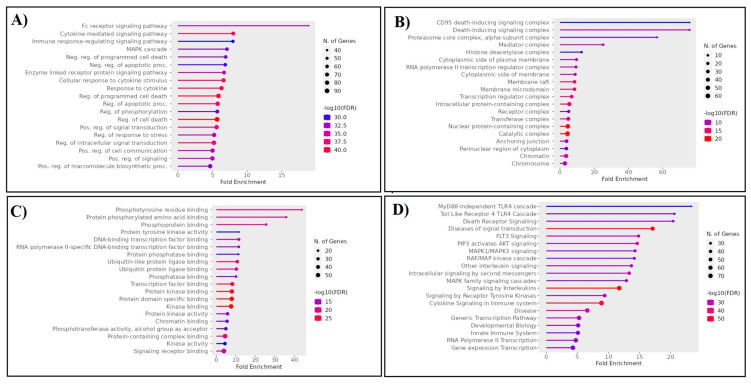
Ontological analysis demonstrates the (**A**) biological process, (**B**) cellular component, (**C**) molecular function, and (**D**) molecular pathways of the proteins in the top five clusters of the protein network.

**Figure 4:**
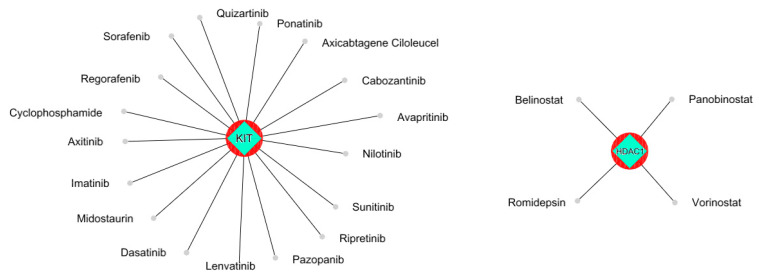
The KIT protein was observed to be a potential target for most of the anti-cancer drugs. HDAC1 was shown to be a target for three anti-cancer drugs.

**Figure 5:**
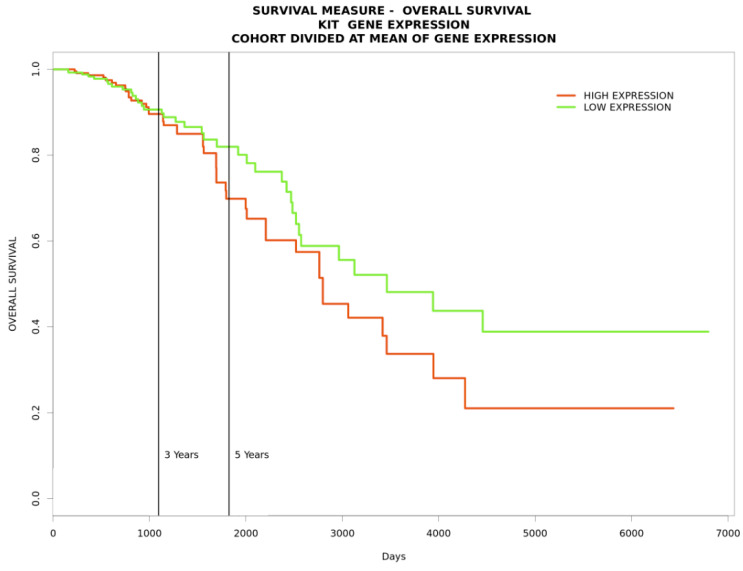
A survival plot indicating that increased KIT expression decreases the survival rate of patients with breast cancer.

To gain further insight into the functional relationships among the DEGs of PBC (GSE205185), we constructed a PBC protein interaction (PPI) network using Cytoscape version 3.9.1 (Figure 2A). Then, the PPI was dissected into multiple clusters using the MCODE algorithm to select the top five clusters (Figure 2B–F). These clusters were observed to be involved in cancer-associated functions based on ontological and pathway analyses (Figure 3A–D). Among the five clusters, the cluster presenting the greatest number of RRbc DEG (GSE210306) encoding proteins was selected. A total of nine (FADD, POLR2A, HIPK2, HDAC1, KIT, CUL4B, ARRB2, FOS, and YWHAH) RRbc DEG-encoding proteins were observed to be distributed among the clusters. Interestingly, cluster-D presents the majority of RRbc proteins (KIT, HDAC1, and CUL48). Further, the cancer drug database was used to screen the proteins in cluster-D for their drug target potential. Notably, the KIT (proto-oncogene c-KIT) protein in cluster-D was observed to be a target for most of the anti-cancer drugs (Figure 4). In our analysis, KIT was observed to be upregulated in both DEGs of primary breast tumors and RRbc. On the other hand, survival analysis showed increased KIT expression, which decreased the survival rate of the patients, with a hazard ratio of 1.09 (0.97–1.21) (Figure 5). Henceforth, the KIT protein was determined to be an “RRbc target” based on four significant properties: Firstly, the KIT protein was influenced in primary breast tumors, identified through cluster analysis; secondly, the KIT protein has radioresistant behavior, demonstrated through DEG analysis of GSE210306; thirdly, the KIT protein is observed as a cancer drug target as assessed through database screening; and finally, KIT expression influences the survival rate of patients with breast cancer.

### 2.3. Phytochemicals and Molecular Docking

**Figure 6:**
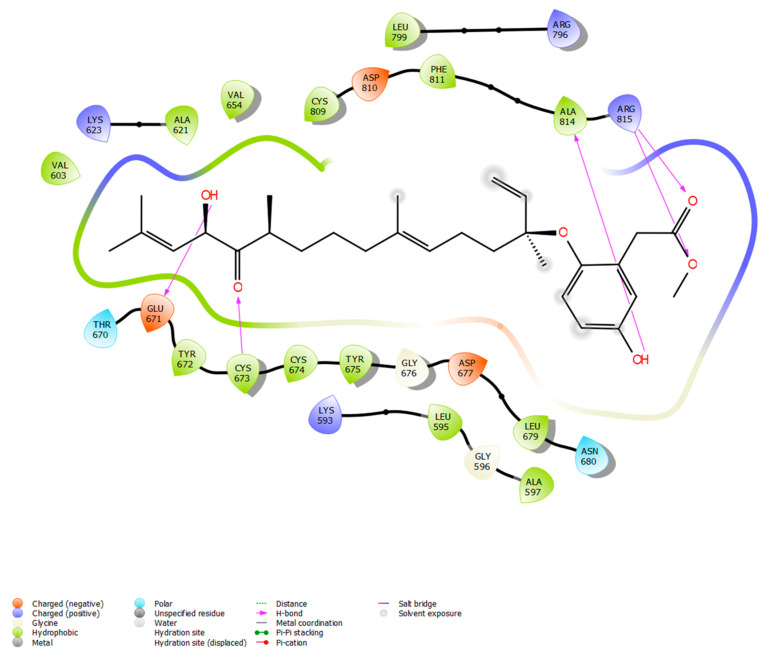
Docking between KIT and nahocol-A1 shows potential binding modes in forming five hydrogen bonds at CYS673, GLU671, ALA814, and ARG815 of the protein-binding site.

Following a search in the Comprehensive Marine Natural Products Database (CMNPD), a set of phytochemicals sourced from brown algae (*n* = 1212) was collected for subsequent molecular docking against the KIT target protein. The phytochemicals were retrieved in SDF format and then optimized using the Ligprep module in the Maestro 11.2 Schrödinger software. Simultaneously, the protein structure and the inhibitor binding sites of the KIT target were acquired from the protein data bank (PDB ID: 3G0E). Then, using the receptor grid generation module, the grid (30 × 30 × 30 A°) around the binding sites (CYS673, GLU671, ALA621, VAL603, CYS809, PHE858, GLY676, CYS674, LEU595, and ASP677) was generated. Further, molecular docking was executed with the Glide module to investigate the binding efficiencies of 1212 phytochemicals with the KIT target. The favorable binding pose was selected based on the Glide score, which reflects the binding affinity between the phytochemicals and the target protein (Table 1). From the 1212 phytochemicals that were docked, the five phytochemicals with the lowest Glide score were chosen. Among the top five, nahocol-A1 had the lowest Glide score (−8.56 kcal/mol) and the most favorable interactions with the KIT target. Notably, nahocol-A1 formed six hydrogen bonds with the critical residues CYS673, GLU671, ALA814, and ARG814 of the binding site, as depicted by the molecular interaction plot (Figure 6). Then these selected phytochemicals were subjected to the QikProp module for ADME screening (Table S1). The QikProp module (Maestro 11.2 version, Schrödinger software) utilized pharmacological and physicochemical properties to screen the molecules and provide a star score value (the lower the star score, the higher the drug-like properties). Herein, the phytochemicals with a star score of less than five were screened to confirm their drug-like properties. Of the top five phytochemicals analyzed, nahocol-A1, 4′-chloro-2-hydroxyaurone, mediterraneol B, and (2E,6E,10E)-1-(2,5-dihydroxy-3-methylphenyl)-13-hydroxy-3,7,11,15-tetramethylhexadeca-2,6,10,14-tetraen-5-one were found to have drug-like properties, with star scores of less than five, with the exception of ishigoside (star score: 12, above our cut-off).

### 2.4. Density Functional Theory of the Selected Compounds

**Figure 7:**
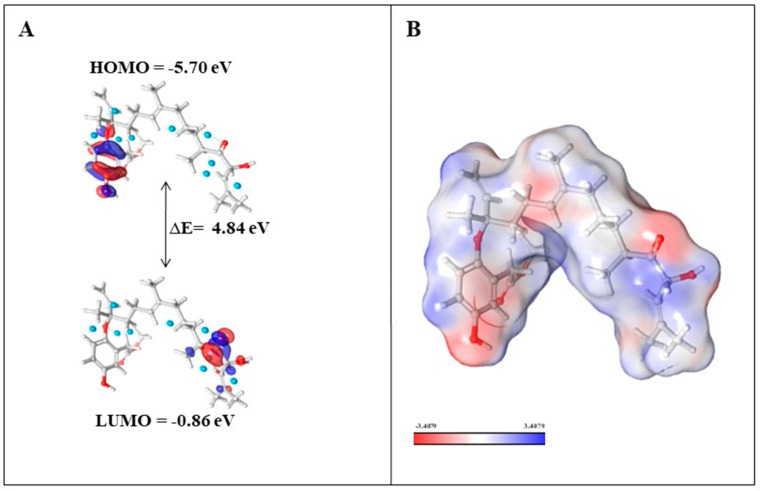
*(***A**) Energy levels of nahocol-A1 at the highest occupied molecular orbital (HOMO) and lowest unoccupied molecular orbital (LUMO); (**B**) molecular electrostatic potential of nahocol-A1.

Density functional theory (DFT) calculations were performed to investigate the electronic and molecular properties for ADME-screened phytochemicals (nahocol-A1, 4′-chloro-2-hydroxyaurone, mediterraneol B, and (2E,6E,10E)-1-(2,5-dihydroxy-3-methylphenyl)-13-hydroxy-3,7,11,15-tetramethylhexadeca-2,6,10,14-tetraen-5-one). The optimized molecular structure and electronic properties were obtained using the B3LYP-D3/6-31G level of theory. Frontier molecular orbitals such as HOMO (Highest Occupied Molecular Orbital) and LUMO (Lowest Unoccupied Molecular Orbital) energies were calculated to evaluate the chemical reactivity and stability of these four compounds. According to the frontier molecular orbital calculation, the energy difference (ΔE) between HOMO and LUMO enhances the stability and reactivity of the compound by promoting intramolecular charge transfer [14]. Also, the large energy gap denotes less reactivity and more stability, while the small energy gap denotes more reactivity and less stability [14]. Among the analyzed phytochemicals (Table 2), a high energy gap was noticed for nahocol-A1 (ΔE = 4.84 eV) (Figure 7A). Such a high energy gap between the HOMO and LUMO energies suggests that nahocol-A1 has high stability and less chemical reactivity. The energy values of the HOMO and LUMO were used to derive the chemical descriptors using mathematical equations (described in the methodology section).

#### 2.4.1. Evaluation of Chemical Descriptors

The quantum chemical descriptors for the ADME-screened phytochemicals are depicted in Table 2. Using mathematical equations, the ionization potential, electron affinity, energy gap, chemical hardness, chemical softness, electronegativity, electrophilicity, and chemical potential were calculated and compared between nahocol-A1, 4′-chloro-2-hydroxyaurone, mediterraneol B, and (2E,6E,10E)-1-(2,5-dihydroxy-3-methylphenyl)-13-hydroxy-3,7,11,15-tetramethylhexadeca-2,6,10,14-tetraen-5-one. Ionization potential (I) implies molecular stability and the energy needed to remove an electron from a molecule’s ground state, and electronic affinity (A) is the energy released when a molecule in the ground state attracts an electron. Correspondingly, nahocol-A1 has good chemical stability with a high ionization potential (I = 5.70 eV) and electronic affinity (A = 0.86 eV). During a reaction, soft molecules change their electronic distribution, and hard molecules rarely perform. Our results revealed that nahocol-A1 has better softness (0.41 eV) and moderate hardness (2.42 eV) values. The electrophilicity index (ω) describes the electrophile nature of the compound, while electronegativity (χ) measures the ability to attract electrons. The order of electrophilicity ranges were as follows: weak electrophiles (ω < 0.8 eV), moderate electrophiles (0.8 < ω < 1.5 eV), and strong electrophiles (ω > 1.5 eV). Table 2 shows that nahocol-A1 was observed to have better stability than other analyzed phytochemicals.

**Table 2:** Quantum chemical descriptors of ADME-screened phytochemicals.

Quantum Chemical Descriptors	Nahocol-A1	4′-Chloro-2-hydroxyaurone	Mediterraneol B	(2E,6E,10E)-1-(2,5-Dihydroxy-3-methylphenyl)-13-hydroxy-3,7,11,15-tetramethylhexadeca-2,6,10,14-tetraen-5-one
	Electron Volt (eV)			
Homo energy (E_HOMO_)	−5.7	−4.81	−4.01	−4.21
Lumo energy (E_LUMO_)	−0.86	−1.6	−1.35	−1.45
Ionization potential (I)	5.7	4.81	4.01	4.21
Electron affinity (A)	0.86	1.6	1.35	1.45
Energy gap (ΔE)	4.84	3.2	2.66	−2.76
Chemical hardness (η)	2.42	1.6	1.33	1.38
Chemical softness (σ)	0.41	0.62	0.75	0.72
Electronegativity (χ)	3.28	3.21	2.6	2.83
Electrophilicity (ω)	2.22	3.04	2.6	3.01
Chemical potential (µ)	−3.28	−3.21	−1.75	−2.83

#### 2.4.2. Molecular Electrostatic Potential

The MEP (molecular electrostatic potential) map was developed for nahocol-A1 to visualize the electrostatic potential distribution of the molecule, hydrogen bonding, and chemical reactivity. MEP illustrates the electron density cloud around the nuclei of the molecule and also describes the hydrogen bonding and non-covalent interactions that are involved in chemical reactivity. The electronegative and electropositive potential regions of nahocol-A1 were represented in red and blue colors (Figure 7B, increasing order of ESP (electrostatic potential) regions: red < white < blue). The red regions are prone to electrophilic attack, whereas the blue regions are favorable sites for nucleophilic attack [15]. Overall, the DFT results suggest that nahocol-A1 has more favorable electronic and molecular properties than other phytochemicals. Hence, nahocol-A1 was assessed for its binding free energy through MM-GBSA and for its stability with the KIT protein through molecular dynamics simulation.

### 2.5. MM-GBSA Calculation of the KIT-nahocol–A1 Complex

The molecular mechanics-generalized Born surface area (MM-GBSA) was assessed to evaluate the binding free energy of the KIT–nahocol-A1 complex. Table 3 establishes the various components of binding energy, such as Gibb’s free energy, hydrogen bonds, generalized Born solvation, lipophilic, columbic, and van der Waals energies. The nahocol-A1 effectively binds (ΔGbind = −53.12 kcal/mol) through hydrogen bonds that contribute significant stability with the KIT protein.

### 2.6. Molecular Dynamics Simulation of the Complex

**Figure 8:**
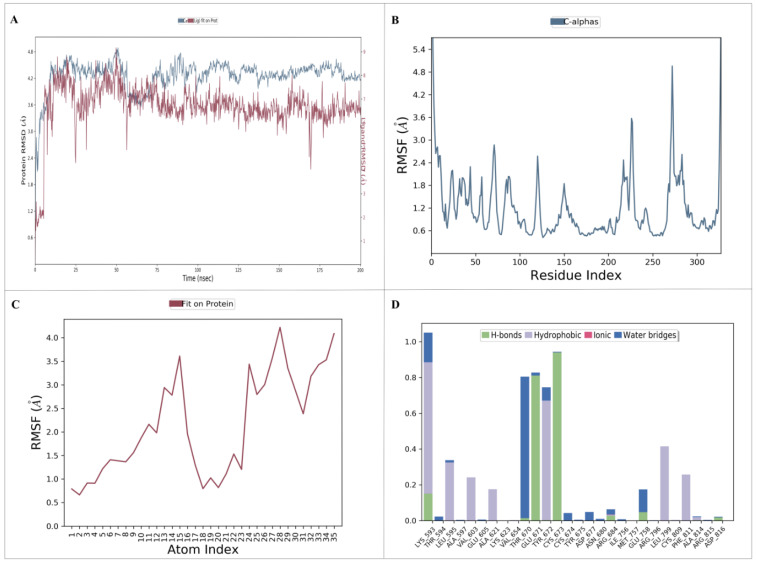
Molecular dynamics simulation provides insights into the dynamic behavior and conformational changes of a biomolecular system over time. (**A**) RMSD plot of the protein–ligand complex; (**B**) RMSF plot of the target protein; (**C**) RMSF plot of the ligand; and (**D**) protein and ligand interaction plot.

A molecular dynamics (MD) simulation was performed to investigate the stability and conformational changes of the KIT–nahocol-A1 complex. The simulation was run for 200 ns, and then the outcome trajectories, such as root mean square deviation (RMSD), root mean square fluctuation (RMSF), and protein–ligand contacts, were analyzed. The RMSD plot is depicted in Figure 8A. The KIT–nahocol-A1 complex showed minimal fluctuation throughout the simulation time. In particular, the KIT–nahocol-A1 complex attains stability between 110 ns and 200 ns. On the other hand, the RMSF plot (Figure 8B) demonstrates the fluctuation in protein residues that occurred due to the binding of nahocol-A1. Notable fluctuations were observed in the backbone residue positions between ALA755 and GLY803 at 1.03 Å. Likewise, ligand RMSF (Figure 8C) showed flexibility at the 13, 15, 24, and 28 atom numbers of nahocol-A1, contributing effective binding to the KIT protein. Further, the protein–ligand interaction (Figure 8D) of the KIT–nahocol-A1 complex exhibits the formation of hydrogen bonds at GLU671, CYS673, and LYS593, hydrophobic interaction at LYS593, LEU595, TYR672, LEU799, and PHE811, and a water bridge at THR670 of the KIT protein.

## 3. Discussion

In spite of extensive experiments and the availability of multiple chemotherapeutic agents, their efficacy in treating breast cancer is still low because of complicated tumor interactions and the resistance of cancer cells to various therapies. Daniel A. Dias et al. demonstrate the revolution from synthetic medicine to traditional medicine in the quest for elucidations of several diseases, particularly cancer [16]. The utilization of natural products in medicine continues to be a significant area of research in healthcare. Natural products, derived from plants, animals, and microorganisms, have provided the basis for numerous pharmaceuticals and therapies, contributing to both traditional and modern medicine [17]. These natural products often contain a wide array of bioactive compounds, such as alkaloids, flavonoids, terpenoids, and polyphenols, which have demonstrated diverse pharmacological activities. Phytochemicals have diverse biological activities and are known for their antioxidant, anti-inflammatory, antimicrobial, and anti-cancer properties [18].

Natural marine-derived compounds have gained significant attention in the field of medicine due to their diverse chemical structures and potential pharmacological activities. These compounds are extracted from various marine organisms, including algae, sponges, corals, and microorganisms, and have shown promise in drug discovery and development [19]. Our study focused on investigating the potential of brown algae-derived compounds in the context of radioresistant breast cancer. Brown algae, a category of seaweed found in marine environments, have gained attention for their rich content of bioactive compounds, some of which may hold promise in the fight against breast cancer. Brown algae, a category of marine seaweeds, are rich sources of bioactive compounds with diverse pharmacological activities. Fucoxanthin, fucoidans, and polyphenols found in brown algae exhibit antioxidant, anti-inflammatory, anti-cancer, and immunomodulatory properties. These compounds have demonstrated potential for inhibiting cancer cell proliferation, modulating the immune system, and promoting cardiovascular health [20]. Based on this background, brown algae-derived phytochemicals were investigated against the radioresistant target protein.

To elucidate the RRBC target, we implemented a series of computational approaches. Our assessment includes gene expression analysis, protein network construction, dissection into multiple clusters, and ontological assessment. As an outcome, the selected top five clusters from the network are crucially involved in the MyD88-independent TLR4 cascade, death receptor signaling, FLT3 signaling, PIP3 activating, AKT signaling, and MAPK1/MAPK3 signaling. These pathways are well known for their involvement in cancer. Furthermore, integrating the RRbc DEGs into the clusters suggests the involvement of radioresistant genes such as *FADD, POLR2A, HIPK2, HDAC1, KIT, CUL4B, ARRB2, FOS,* and *YWHAH* as an integral part of the analyzed clusters. For instance, FADD plays a significant role in cancer metastasis, the spread of cancer cells to distant organs. Dysregulated FADD expression may contribute to the survival and migration of cancer cells during metastasis [21]. Likewise, POLR2A overexpression has been associated with tumor progression and a poor prognosis in several cancer types. Also, POLR2A contributes to the activation of cell proliferation, angiogenesis, and metastasis [22]. Similarly, HIPK2 plays a role in the cellular response to DNA damage, and its expression levels can serve as prognostic markers for certain cancers [23]. HDAC1 is involved in various cellular functions, and its dysregulation has been implicated in cancer development and progression. Altered histone acetylation patterns in HDAC1 can result in epigenetic changes that contribute to cancer initiation and progression [24]. In cancer, the presence of KIT mutations has prognostic significance, helping to predict disease behavior and patient outcomes. CUL4B is a member of the Cullin–RING E3 ubiquitin ligase complex, which is involved in the ubiquitin–proteasome system and linked to the regulation of the cell cycle. Altered expression of CUL4B has been reported in various types of cancer, including breast, lung, liver, and ovarian cancers [25]. ARRB2 is a protein that is involved in regulating G-protein-coupled receptor (GPCR) signaling, which plays a critical role in cell communication and various physiological processes. ARRB2 can influence signaling pathways downstream of GPCRs, which are involved in cellular responses such as proliferation, migration, and survival [26]. Dysregulation of these pathways can contribute to cancer development and progression. FOS is a proto-oncogene that promotes cell growth and proliferation by regulating the expression of genes involved in the cell cycle. Overexpression of c-Fos has been associated with increased cell proliferation, a characteristic of cancer cells [27]. Among the five clusters, cluster-D was noticed to have three RRbc genes. Following the selection of cluster-D, these proteins were assessed for their behavior as anti-cancer drug targets. Among the three RRbc proteins in cluster-D, KIT was observed to be a frequent target for most of the anti-cancer drugs and an influence on the survival rate.

*KIT* is a proto-oncogene receptor tyrosine kinase, localized at chromosome position 4q11-q12, that encodes the receptor tyrosine kinase protein. *KIT* is involved in a variety of biological processes, which include activating its cytokine ligand, stem cell factor (SCF). This protein phosphorylates multiple intracellular proteins that play a role in the proliferation, differentiation, migration, and apoptosis of many cell types, and thereby plays an important role in hematopoiesis, stem cell maintenance, gametogenesis, melanogenesis, and mast cell development, migration, and function [28]. Notably, *KIT* was considered a potential drug target [29] for gastrointestinal stromal tumors, melanoma, and acute myeloid leukemia that was inhibited by imatinib, tandutinib, sunitinib, and dasatinib. However, these drugs were observed to have resistance and were reported to have serious cardiotoxicity [16]. Wragg et al. indicate that sunitinib is an effective drug for metastatic breast cancer patients but acquires innate drug resistance and enhances metastasis [30]. Thus, we looked for natural compounds that may inhibit the selected radioresistant target. Henceforth, the phytochemicals of brown algae were retrieved from the database and implemented for molecular docking to analyze their binding affinity with the KIT protein. Based on molecular docking, nahocol-A1 was observed to have the least binding energy (−8.56 kcal/mol), which contributes to its high affinity with the KIT protein. Simultaneously, ADME analysis of nahocol-A1 showed high drug-like properties based on a star score with zero violations on the basis of Lipinski’s rule of five. Then molecular electrostatic potential distribution of nahocol-A1 was analyzed to describe hydrogen bonding and non-covalent interactions that exhibit good chemical stability through DFT examination. Further, the MM-GBSA was calculated for the KIT–nahocol-A1 complex, and the binding energy (−53.12 kcal/mol) showed a significant mode of binding affinity and confirmed the overall stability of the complex. Henceforth, nahocol-A1 was considered for MD simulation to investigate the conformational stability of the KIT protein.

Based on the RMSD trajectory, the KIT–nahocol-A1 complex was found to have stable conformation throughout the simulation. Also, the protein RMSF defines the residual flexibility of a protein and allows the binding of nahocol-A1. Further protein–ligand interaction plots revealed that nahocol-A1 formed stable hydrogen bonds with GLU671, CYS673, and LYS593, hydrophobic interaction at LYS593, LEU595, TYR672, LEU799, and PHE811, and a water bridge at THR670 of the KIT protein. Our study elucidates that several quantum chemical descriptors are known to influence protein–ligand interactions. Thus, the number of hydrogen donors in ligands is one of the key factors influencing their binding affinities with target proteins. Nahocol-A1 has a higher capacity to form hydrogen bonds and tends to exhibit strong interaction with the KIT protein, potentially leading to its use as a drug candidate. Additionally, to evaluate the mechanism of active sites during protein–ligand interactions, it offers the chemical correctness of the drug molecules. Thus, in DFT analysis, frontier molecular orbitals such as HOMO (−5.70 eV) and LUMO (−0.86 eV) estimated the chemical reactivity of the nahocol-A1 complex with good electronic properties, which were in agreement with the outcome of molecular docking and dynamic simulation evaluation. Henceforth, our sequential analyses, such as DEGs of radioresistant and primary breast cancer, protein interaction networks, target selection from the cluster, phytochemical screening through ADME and molecular docking, complex assessment with quantum chemical analysis, MM-GBSA, and molecular dynamics (MD) simulation, allow us to validate the molecular behavior of RRbc and identify a candidate molecule derived from brown algae.

## 4. Materials and Methods

### 4.1. Data Collection and Differential Gene Expression Analysis

The gene expression datasets were searched independently by the two authors in the Gene Expression Omnibus (https://www.ncbi.nlm.nih.gov/geo/, accessed on 13 May 2023) using a variety of keywords relating to (1) primary breast cancer, (2) breast cancer radiotherapy, and (3) recurrent breast cancer. Then the collected datasets were assessed based on the following criteria: the inclusion criteria were (a) gene expression executed in the microarray platform; (b) a dataset containing a minimum of three samples in each group to apply relevant statistical analyses; and (c) a dataset with an appropriate comparative group. Likewise, exclusion criteria include (a) studies with non-Homo sapiens as well as in vivo; (b) a dataset with less than two samples in a group; (c) studies without relevant clinical or molecular confirmation for sample classification; and (d) microarray expression other than mRNA. Finally, the selected datasets were confirmed through group discussion with the other authors. Notably, the GSE205185 dataset that was selected, which consists of primary breast tumor (PBC) tissue, contains 22 samples: 17 patients with primary breast tumor tissue and five healthy individuals. Similarly, the GSE210306 dataset was included in our analysis and contains four RRbc and four non-radioresistant (parental) samples. Each dataset was subjected to the Limma package (R-program) to identify differentially expressed genes (DEGs). For instance, the DEGs for GSE205185 were determined by comparing the normal and PBC tissues. Likewise, the DEGs for GSE210306 were extracted by comparing RRbc vs. parental tissue. The DEGs from each dataset were selected based on the log fold change >2 with an adj *p* < 0.05 [31].

### 4.2. Protein Network Construction and Cluster Analysis

To construct the protein–protein interaction (PPI) network, the DEGs obtained from the analysis of GSE205185 were subjected to the Gene mania plug-in, Cytoscape software version 3.9.1 [32]. In the constructed network, nodes depict proteins, and the edges represent their interactions. Using the Cytoscape MCODE plug-in, the entire protein network was dissected to have multiple hubs called clusters that describe the core functional components of the PBC network with highly interconnected edges [33]. From the generated clusters, the top five clusters were selected and subjected to ShinyGO (http://bioinformatics.sdstate.edu/, accessed on 26 June 2023) for ontological and pathway analysis. ShinyGO is a graphical web tool that provides large annotations for the inputted protein list to evaluate their functional importance, such as gene ontology (molecular function, biological processes, and cellular components) and molecular pathway [34].

### 4.3. Screening of Therapeutic Targets and Phytochemicals

Next, the top five clusters were searched for the presence of any RRbc DEG-encoding protein derived from the GSE210306 dataset. The cluster presenting the maximum number of radioresistant protein-encoding genes was selected. The selected cluster represents the behavior of breast cancer as well as its radioresistant properties. Further, the cancer drug database (https://www.anticancerfund.org/en/cancerdrugs-db, accessed on 6 July 2023) was utilized to assess the cluster proteins for their potential as cancer targets with known anti-cancer drugs. Additionally, gene expression-based survival analysis was performed using the Progtool web server (http://www.progtools.net/, accessed on 23 July 2023) with the TCGA-BRACA dataset to confirm the significance of a chosen gene versus the survival rate based on its expression.

### 4.4. Comprehensive Marine Database Ligand Preparation

The Comprehensive Marine Natural Products Database (CMNPD) (https://www.cmnpd.org, accessed on 3 August 2023) was screened to identify the potential phytochemicals for the “RRbc target”. A total of 1212 compounds were retrieved from the source of brown algae. All the compounds were downloaded in the SDF format; further, these compounds were prepared using Ligprep (Maestro v11.2, Schrödinger suite) for molecular docking. In the process of ligand preparation, the hydrogen atoms were added, and the generation of various ionization states and tautomers and optimization of the geometries were prepared using the Epik configuration. Further, all the ligands were optimized by the OPLS3 force field for subsequent processes [35].

### 4.5. Protein Preparation and Molecular Docking

Simultaneously, the protein structure of the “RRbc target” was retrieved from the Protein Data Bank (PDB ID: 3G0E) and optimized with the OPLS3 force field using the protein preparation wizard, Maestro 11.2 version, and Schrödinger suite [36]. Further, the protein structure of the “RRbc target” was subjected to a Glide receptor grid generation panel to generate a three-dimensional (3D) grid box around the active site residues. The active sites for the “radioresistance target” were acquired from PDB ID 3G0E. Finally, the docking was performed using the Glide module, Maestro 11.2 version, Schrödinger, to evaluate the binding affinity based on the Glide score for each phytochemical against the RRbc target.

### 4.6. ADME Parameters

The QikProp module, Maestro 11.2 version, Schrödinger, was used to evaluate the ADME properties of the selected top five phytochemicals based on their Glide score. The QikProp module provides a star rating for each phytochemical based on ADME descriptors. Overall, fifty ADME descriptors were assessed, including cell permeability (pCaco-2 and pMDCK) and oral absorption (% human oral absorption), providing a star score; a lower number of stars indicates high drug-like properties [37]. In our case, the phytochemicals with a star score of less than five were selected for subsequent analysis.

### 4.7. Density Functional Theory

Density functional theory (DFT) was implemented to investigate the atomic and molecular properties of the selected phytochemicals with an ADME star score of less than five. The 3D structure of the phytochemicals was optimized at the B3LYP-D3/6-31G* level of theory using the Jaguar module, Maestro 11.2 version, Schrödinger. DFT energetics establish 3D electronic states of the phytochemicals to determine the transfer of lone pairs, bonds, and chemical reactivity [14]. Further, the optimized phytochemical structure was examined based on quantum chemical descriptors, such as the highest occupied molecular orbital (HOMO), lowest unoccupied molecular orbital (LUMO), molecular electrostatic potential (MEP), frontier molecular orbital chemical stability, and donor–acceptor interactions. The energy gap between the HOMO and LUMO indicates the kinetic stability of phytochemical and intramolecular charge transfer. Molecular electrostatic potential depicts the electron density distribution and chemical reactivity around the analyzed phytochemical. The mathematical formulations that define these descriptors can be described as follows [38,39,40]:

$$Ionization\;potential\;\left(I\right)=-E_{HOMO}$$

$$Electron\;affinity\;\left(A\right)=-E_{LUMO}$$

$$Energy\;gap\;\left(\Delta E\right)=E_{LUMO}-E_{HOMO}$$

$$Chemical\;hardness\;\left(\eta \right)=\frac{I-A}{2}$$

$$Chemical\;softness\;\left(\sigma \right)=\frac{1}{\eta }$$

$$Electronegativity\;\left(\chi \right)=\frac{I+A}{2}$$

$$Electrophilicity\;index\;\left(\omega \right)=\frac{\mu^{2}}{2\eta }$$

$$Chemical\;potential\;\left(\mu \right)=-\frac{\left(I+A\right)}{2}$$

### 4.8. Prime MM-GBSA Calculation

Molecular mechanics-generalized Born surface area (MM-GBSA) is a significant approach to estimating the binding free energy of the protein–ligand complex [41,42]. In addition, this approach is useful in the validation of docking results to remove significant false positives in the protein–ligand interaction. Herein, the MM-GBSA prime module of Schrödinger Maestro 11.2 was used to evaluate the binding free energy of the selected phytochemical with its protein target.

### 4.9. Molecular Dynamics Simulation

The Desmond program [43], Schrödinger suite 2019 version, was used to perform MD simulation to validate the conformational changes of the protein structure of the “RRbc target” upon binding of selected phytochemicals. The system builder panel was employed to generate an orthorhombic box and solvated with a simple point charge (SPC) water model. Further, the system was neutralized by adding an adequate number of Na^+^ or Cl- ions. Here, we have added single Cl- ions to neutralize our system. Then, energy minimization was performed, and MD simulation was executed for 200 ns with a recording interval of 200 ps. The NPT ensemble was kept at a constant temperature of 300 K and 1 bar of pressure throughout the simulation [44]. The outcome of the simulation was analyzed based on the trajectory of root mean square deviation (RMSD), root mean square fluctuation (RMSF), and protein–ligand contacts.

## 5. Conclusions

Within the context of conventional medicine and therapeutic possibilities, it is of the utmost significance to develop an effective treatment against radioresistant breast cancer. This is one of the most pressing medical challenges of our time. On the other hand, it has been shown that the utilization of natural plants has the potential to halt the progression of this complex disease. The major purpose of this investigation was to recognize a selection of phytochemicals that were found in brown algae. The molecule nahocol-A1 showed the most promising binding affinity against the KIT protein, which is the primary target, according to our analysis. The findings provide credibility to the concept that the phytochemicals that are produced by brown algae have the potential to be employed as a therapy for breast cancer. It is feasible that the accuracy of these findings might be investigated further through the use of experimental research on animal models, which would pave the way for translational medicine to treat breast cancer with radioresistant properties.

## Figures and Tables

**Table 1:** Docking scores of the top five phytochemicals against the KIT protein.

SI. No	Compounds	Glide Score (kcal/mol)
1	Nahocol-A1	−8.56
2	Ishigoside	−8.53
3	4′-chloro-2-hydroxyaurone	−8.51
4	Mediterraneol B	−8.46
5	(2E,6E,10E)-1-(2,5-dihydroxy-3-methylphenyl)-13-hydroxy-3,7,11,15-tetramethylhexadeca-2,6,10,14-tetraen-5-one	−8.31

**Table 3:** MM-GBSA calculation for the KIT–nahocol-A1 complex.

Complex	Δgbind (eV)	Δgcoul (eV)	ΔGH-Bond (eV)	Δglipo (eV)	ΔGGB (eV)	ΔGvdW (eV)
KIT–nahocol-A1	−53.12	−32.92	−3.58	−18.01	−31.47	−40.54

## Data Availability

The data used to support the findings of this study are included within the article.

## Supplementary Materials

The following supporting information can be downloaded at: https://www.mdpi.com/article/10.3390/md21100545/s1. Table S1. ADMET properties of nahocol-A1 assessed through the QikProp module, Maestro 11.2 version, Schrödinger.
